# Continuous cow’s milk protein ingestion during infancy may promote casein-specific IgG4 production

**DOI:** 10.1016/j.jacig.2024.100257

**Published:** 2024-04-10

**Authors:** Tetsuhiro Sakihara, Kenta Otsuji, Yohei Arakaki, Kazuya Hamada, Shiro Sugiura, Komei Ito

**Affiliations:** a Department of Pediatrics, Heartlife Hospital, Okinawa, Japan; b Department of Pediatrics, Okinawa Kyodo Hospital, Okinawa, Japan; c Department of Pediatrics, Naha City Hospital, Okinawa, Japan; d Department of Child Health and Welfare (Pediatrics), Graduate School of Medicine, University of the Ryukyus, Okinawa, Japan; e Department of Allergy, Aichi Children’s Health and Medical Center, Aichi, Japan

**Keywords:** Food allergy, milk allergy, early introduction, randomized controlled trial, IgG4, infant formula, cow’s milk formula, prevention, sensitization

## Abstract

**Background:**

Early supplementation and subsequent discontinuation of cow’s milk formula (CMF) may increase the risk of cow’s milk allergy in breast-fed infants, but little is known about the relationship between continuous CMF ingestion and cow’s milk protein–specific immunoglobulin production.

**Objective:**

This study aimed to clarify the aforesaid relationship in cow’s milk–sensitized infants.

**Methods:**

Using data from a randomized controlled trial of a Japanese birth cohort, we performed a subgroup analysis of participants who had ingested CMF in the first 3 days of life and exhibited a positive skin prick test response to cow’s milk at age 6 months. We compared the differences in median titers of cow’s milk–specific IgE, casein-specific IgE, and casein-specific IgG4 levels between participants who continued daily or intermittent CMF ingestion up to age 6 months (the “continuous group”) and participants who discontinued CMF ingestion before age 6 months (the “discontinued group”).

**Results:**

From among 462 trial participants, 49 (10.6%) were included in this study (21 in the continuous group and 29 in the discontinued group). The median titer of cow’s milk–specific IgE was 0.17 kUA/L (interquartile range [IQR] = <0.10 to 0.57) in the continuous group and 0.66 kUA/L (IQR = 0.49-1.18) in the discontinued group (*P* = .0008). The median titer of casein-specific IgE was <0.10 kUA/L (IQR = <0.10 to 0.15) in the continuous group and <0.10 kUA/L (IQR = <0.10 to 0.37) in the discontinued group (*P* = .51). The median titer of casein-specific IgG4 was 2.58 mg_A_/L (IQR = 0.77-6.73) in the continuous group and 0.09 mg_A_/L (IQR = 0.07-0.13) in the discontinued group (*P* < .0001).

**Conclusion:**

Continuous CMF ingestion may promote casein-specific IgG4 production in cow’s milk–sensitized infants.

## Introduction

IgE-mediated cow’s milk allergy (CMA) is a common food allergy in infants. We have previously reported the results of the Strategy for Prevention of Milk Allergy by Daily Ingestion of Infant Formula in Early Infancy (SPADE) study, which indicated that the continuous daily ingestion of at least 10 mL of cow’s milk formula (CMF) between 1 and 2 months of age can prevent CMA development.^1^ A subsequent subgroup analysis of the SPADE study found that the early discontinuation of CMF ingestion may increase CMA risk in infants who received CMF in the first 3 days of life.^2^ However, whether continuous CMF ingestion affects cow’s milk protein–specific immunoglobulin production in sensitized infants is unclear. In this new SPADE study subgroup analysis, we examined the relationship between continuous CMF ingestion and cow’s milk protein–specific immunoglobulin production in infants who had ingested CMF in the first 3 days of life and exhibited a positive skin prick test (SPT) response to cow’s milk at age 6 months.

We compared the differences in median titers of cow’s milk–specific IgE, casein-specific IgE, and casein-specific IgG4 between participants who continued daily or intermittent CMF ingestion up to age 6 months (the “continuous group”) and participants who discontinued CMF ingestion before age 6 months (the “discontinued group”) (see the Supplementary Methods in the Online Repository at www.jaci-global.org).

## Results and discussion

Between January 2017 and August 2019, a total of 518 infants were enrolled in the SPADE study, of whom 504 were randomly allocated to an ingestion group or avoidance group (see Fig E1 in the Online Repository at www.jaci-global.org).^1^ From among those participants, we identified 49 who had ingested CMF in the first 3 days of life and had a positive SPT response to cow’s milk at age 6 months. These participants were categorized into this study’s continuous group (n = 21 [7 in the ingestion group and 14 in the avoidance group]) or discontinued group (n = 28 [4 in the ingestion group and 24 in the avoidance group]). Despite being allocated to the SPADE study’s avoidance group, 14 participants were included in the continuous group because their daily CMF consumption records indicated low-dose or intermittent ingestion during the intervention period.

The participants’ characteristics are presented in Table E1 (available in the Online Repository at www.jaci-global.org). There were no significant intergroup differences in baseline characteristics; eczema; or sensitization to egg white, wheat, and soy. There were also no significant intergroup differences in mean wheal diameter in the egg white, wheat, and soy SPT results. However, the continuous group had a significantly higher proportion of paternal atopic diseases and a lower proportion of breast-feeding at age 6 months.

**Fig 1:**
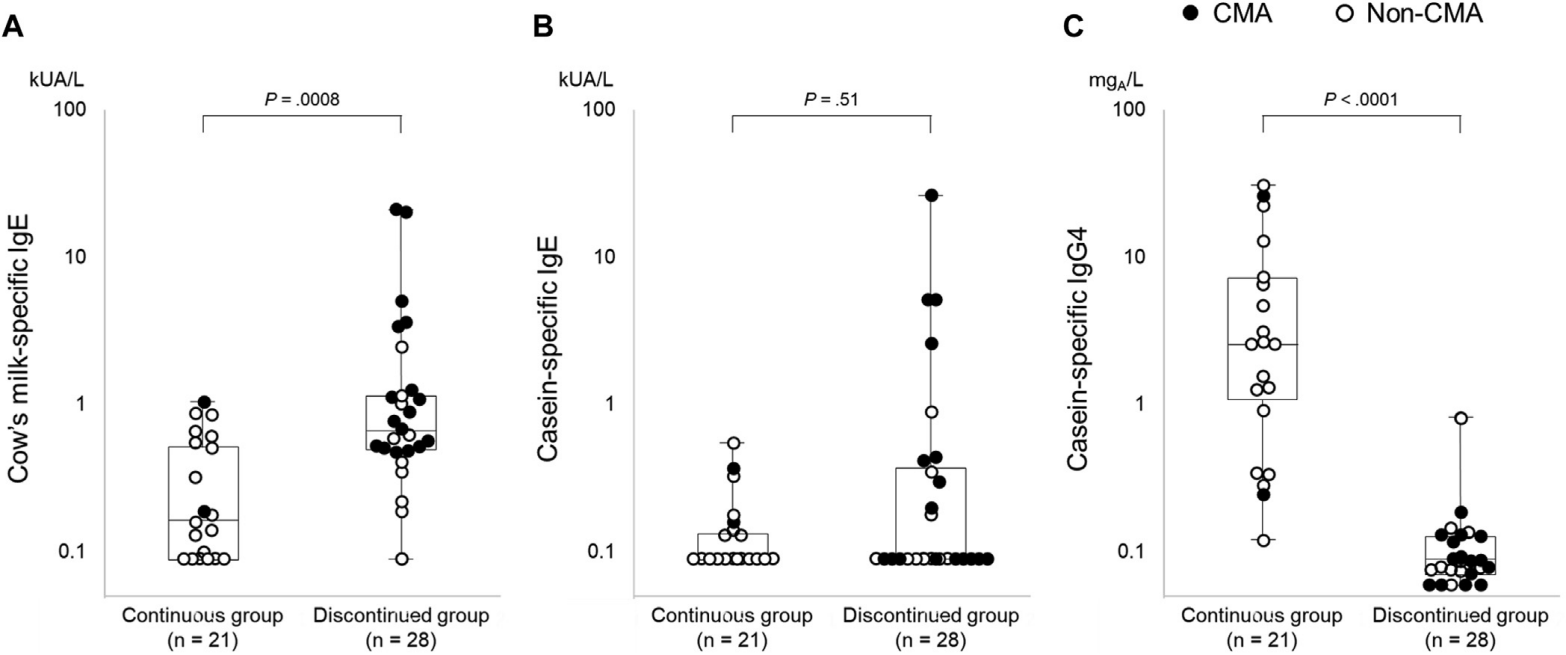
Serum titers of cow’s milk–specific IgE, casein-specific IgE, and casein-specific IgG4 in cow’s milk–sensitized infants at 6 age months. Serum titers of cow’s milk–specific IgE (**A**), casein-specific IgE (**B**), and casein-specific IgG4 (**C**) were measured in participants with cow’s milk sensitization confirmed by an SPT. *P* values were calculated by using the Mann-Whitney *U* test.

The continuous group had significantly lower cow’s milk–specific IgE titers and significantly higher casein-specific IgG4 titers and casein-specific IgG4–to–casein-specific IgE ratios than the discontinued group did (Fig 1 and Table I). CMA incidence in the continuous group (9.5% [2 of 21]) was significantly lower (*P* = .0007) than that in the discontinued group (60.7% [17 of 28]). The clinical details of the participants with CMA are presented in Table E2 (available in the Online Repository at www.jaci-global.org). The relatively low levels of cow’s milk sensitization (IgE titer and wheal diameter) in these participants may be due to the early evaluation (6 months) and inclusion of mild symptoms (eg, localized urticaria) in the CMA definition. None of the participants discontinued CMF ingestion because of suspected CMA symptoms. The continuous group and discontinued group were further subdivided to evaluate differences between participants with and without CMA. In the discontinued group, participants without CMA had a significantly higher frequency and larger volume of CMF ingestion than participants with CMA did (Table I). We found no significant differences in the median titer of casein-specific IgG4 or casein-specific IgG4–to–casein-specific IgE ratio between participants with and without CMA.

**Table I:** Frequency and volume of CMF ingestion, wheal diameter in cow’s milk SPTs, and serum titers of cow’s milk protein–specific immunoglobulins in the continuous and discontinued groups

Characteristic, median (IQR)	All participants (n = 49)			Continuous group (n = 21)			Discontinued group (n = 28)		
	Continuous (n = 21)	Discontinued (n = 28)	*P* value	CMA-positive (n = 2)	CMA-negative (n = 19)	*P* value	CMA-positive (n = 17)	CMA-negative (n = 11)	*P* value
Total number of CMF ingestion days until age 6 mo of age	124 (101-168)	10.5 (4.8-29.3)	<.0001	67 (49-85)	132 (104-169)	.17	5 (3-12)	29 (10-38)	.01
Total volume of CMF ingestion until age 6 mo (mL)	17680 (15,930 to ≥20,000)	716 (177-1,450)	<.0001	10064 (6,624-13,503)	18396 (16,080 to ≥20,000)	.21	305 (106-794)	1423 (385-4,127)	.043
Mean diameter of wheals formed during the cow’s milk SPTs (mm)	2.0 (1.0-3.0)	3.0 (1.5-5.0)	.01	2.5 (2.3-2.8)	2.0 (1.0-2.8)	.39	4.0 (2.0-6.0)	3.0 (1.0-3.0)	.17
Titer of cow’s milk–specific IgE (kUA/L)	0.17 (<0.10 to 0.57)	0.66 (0.49-1.18)	.0008	0.62 (0.40-0.83)	0.15 (<0.10 to 0.54)	.18	0.90 (0.53-3.39)	0.41 (0.21-0.82)	.02
Titer of casein-specific IgE (kUA/L)	<0.10 (<0.10 to 0.15)	<0.10 (<0.10 to 0.37)	.51	0.27 (0.21-0.32)	<0.10 (<0.10 to 0.13)	.055	<0.10 (<0.10 to 0.44)	<0.10 (<0.10 to 0.14)	.20
Titer of casein-specific IgG4 (mg_A_/L)	2.58 (0.77-6.73)	0.09 (0.07-0.13)	<.0001	13.2 (6.74-19.7)	2.58 (1.00-6.05)	>.99	0.09 (0.07-0.12)	0.08 (0.08-0.12)	.98
Casein-specific IgG4–to–casein-specific IgE ratio	14.6 (7.7-74.7)	0.67 (0.37-0.88)	<.0001	82.2 (41.4-123.0)	14.6 (9.11-67.2)	>.99	0.67 (0.17-0.91)	0.84 (0.48-0.87)	.38

*P* values were calculated by using the Mann-Whitney *U* test.

*IQR,* Interquartile range.

**Table II:** Frequency and volume of CMF ingestion, wheal diameter in cow’s milk SPTs, and serum titers of cow’s milk protein–specific immunoglobulins in participants with and without CMA

Characteristic, median (IQR)	All participants (n = 49)			CMA-positive (n = 19)			CMA-negative (n = 30)		
	CMA+ (n = 19)	CMA-(n = 30)	*P* value	Continuous (n = 2)	Discontinued (n = 17)	*P* value	Continuous (n = 19)	Discontinued (n = 11)	*P* value
Total number of CMF ingestion days until age 6 mo	6 (3.5-15.5)	98 (30-137)	<.0001	67 (49-85)	5 (3-12)	.03	132 (104-169)	29 (10-38)	<.0001
Total volume of CMF ingestion until 6 age mo (mL)	358 (121-988)	15542 (3,719 to ≥20,000)	<.0001	10064 (6,624-13,503)	305 (106-794)	.03	18396 (16,080 to ≥20,000)	1423 (385-4,127)	<.0001
Mean diameter of wheals formed during the cow’s milk SPTs (mm)	4.0 (2.0-6.0)	2.0 (1.0-3.0)	.009	2.5 (2.3-2.8)	4.0 (2.0-6.0)	.38	2.0 (1.0-2.8)	3.0 (1.0-3.0)	.23
Titer of cow’s milk–specific IgE (kUA/L)	0.90 (0.53-2.33)	0.22 (<0.10 to 0.61)	.0002	0.62 (0.40-0.83)	0.90 (0.53-3.39)	.35	0.15 (<0.10 to 0.54)	0.41 (0.21-0.82)	.11
Titer of casein-specific IgE (kUA/L)	0.16 (<0.10 to 0.43)	<0.10 (<0.10 to 0.13)	.045	0.27 (0.21-0.32)	<0.10 (<0.10 to 0.44)	.73	<0.10 (<0.10 to 0.13)	<0.10 (<0.10 to 0.14)	>.99
Titer of casein-specific IgG4 (mg_A_/L)	0.09 (0.07-0.13)	0.86 (0.11-2.78)	.004	13.2 (6.74-19.7)	0.09 (0.07-0.12)	.03	2.58 (1.00-6.05)	0.08 (0.08-0.12)	<.0001
Casein-specific IgG4–to–casein-specific IgE ratio	0.67 (0.25-0.96)	9.01 (0.86-28.61)	.0009	82.2 (41.4-123)	0.67 (0.17-0.91)	.14	14.6 (9.11-67.2)	0.84 (0.48-0.87)	<.0001

*P* values were calculated using the Mann-Whitney *U* test.

*IQR,* Interquartile range.

Among the participants with cow’s milk sensitization, those with CMA had higher cow’s milk–specific and casein-specific IgE titers, but lower casein-specific IgG4 titers and casein-specific IgG4–to–casein-specific IgE ratios than those without CMA did (Table II). In all participants with and without CMA, we carried out *post hoc* analyses to compare cow’s milk protein–specific immunoglobulin production between the continuous group and the discontinued group. The median titer of casein-specific IgG4 was significantly higher in the continuous group.

**Table III:** Serum titers of cow’s milk protein–specific immunoglobulins according to SPADE study group allocation

Variable, median (IQR)	All participants (n = 49)			Continuous (n = 21)			Discontinued (n = 28)		
	Ingestion (n = 11)	Avoidance (n = 38)	*P* value	Ingestion (n = 7)	Avoidance (n = 14)	*P* value	Ingestion (n = 4)	Avoidance (n = 24)	*P* value
Titer of cow’s milk–specific IgE (kUA/L)	0.51 (0.15-0.58)	0.55 (0.19-1.09)	.20	0.51 (0.15-0.58)	0.13 (<0.10 to 0.32)	.40	0.38 (0.19-0.59)	0.80 (0.51-1.56)	.10
Titer of casein-specific IgE (kUA/L)	<0.10 (<0.10 to 0.13)	<0.10 (<0.10 to 0.35)	.40	0.13 (<0.10 to 0.16)	<0.10 (<0.10 to 0.14)	.50	<0.10 (<0.10 to <0.10)	<0.10 (<0.10 to 0.43)	.11
Titer of casein-specific IgG4 (mg_A_/L)	2.62 (0.45-10.5)	0.13 (0.08-0.46)	.047	3.12 (2.62-17.7)	1.26 (0.34-4.67)	.046	0.08 (0.07-0.08)	0.09 (0.07-0.13)	.38
Casein-specific IgG4–to–casein-specific IgE ratio	13.4 (3.10-114.6)	0.86 (0.43-5.10)	.007	28.6 (13.4-190.9)	10.2 (3.72-51.9)	.10	0.88 (0.77-0.93)	0.67 (0.29-0.88)	.30

*P* values were calculated using the Mann-Whitney *U* test.

*IQR,* Interquartile range.

We compared cow’s milk protein–specific immunoglobulin production between participants from the SPADE study’s ingestion group and those from the study’s avoidance group after they had been assigned to the continuous group and discontinued group (Table III). In the continuous group, there were no significant differences in the median titers of cow’s milk–specific IgE and casein-specific IgE between the ingestion and avoidance groups. However, the median titer of casein-specific IgG4 was significantly higher in the ingestion group.

**Fig 2:**
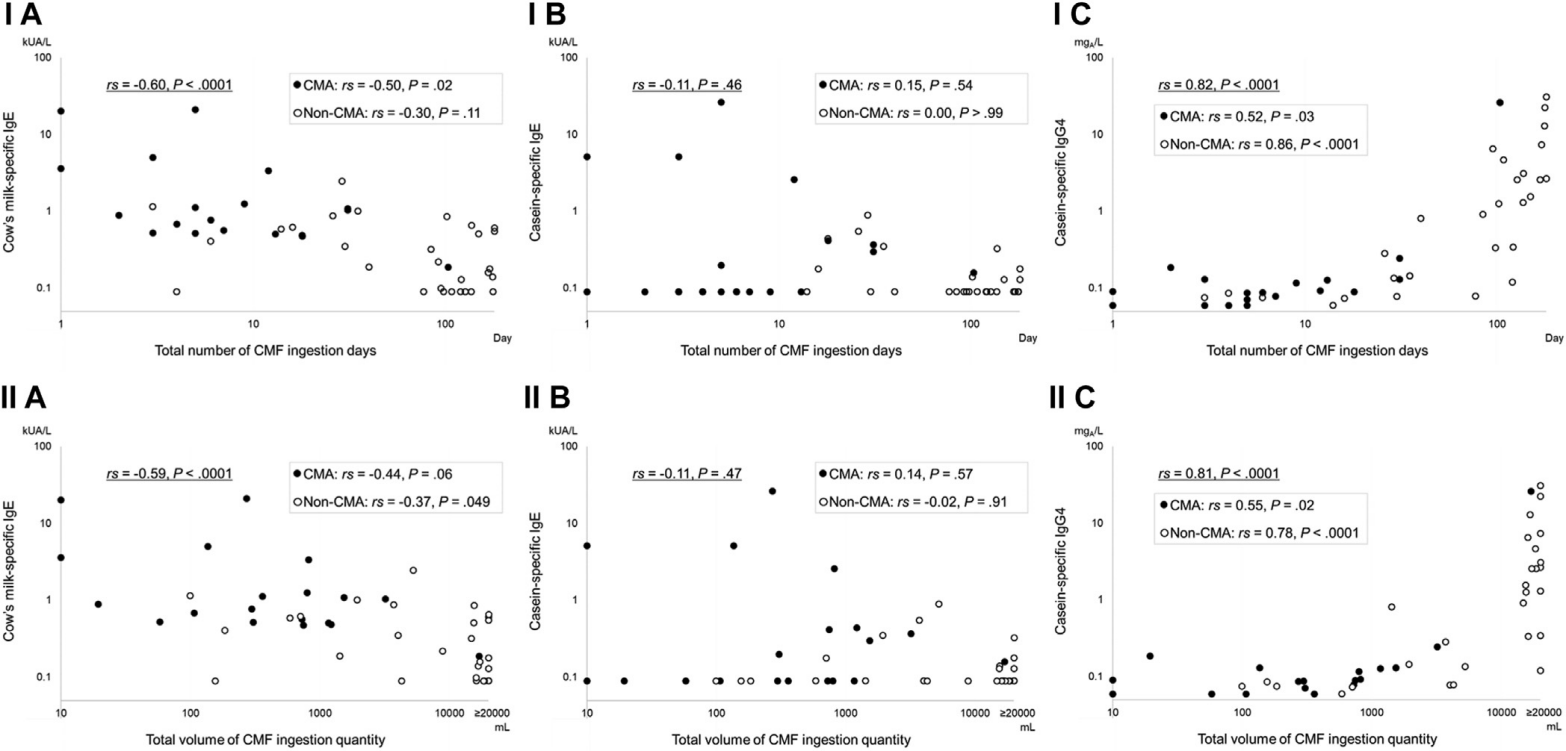
Correlations of the frequency and volume of CMF ingestion with serum titers of cow’s milk–specific IgE, casein-specific IgE, and casein-specific IgG4. Correlations of (**I**) the total number of CMF ingestion days and (**II**) the total volume of CMF ingestion quantity until age 6 months with serum titers of cow’s milk–specific IgE (**A**), casein-specific IgE (**B**), and casein-specific IgG4 (**C**). *P* values were calculated by using the Spearman rank correlation coefficient (significance level .05).

Among all participants in the continuous group (n = 21) and discontinued group (n = 28, including 2 without casein-specific IgG4 titer measurements), both the frequency and volume of CMF ingestion until age 6 months were negatively correlated with cow’s milk–specific IgE titer (*r*_*s*_ = –0.60 and –0.59, respectively) and positively correlated with casein-specific IgG4 titer (*r*_*s*_ = 0.82 and 0.81, respectively) (Fig 2).

We evaluated the correlations between mean wheal diameter in cow’s milk SPTs and serum titers of cow’s milk–specific IgE, casein-specific IgE, and casein-specific IgG4 in the 21 continuous group participants and 28 discontinued group participants (including 2 without casein-specific IgG4 titer measurements). Cow’s milk–specific IgE titer (*r*_*s*_ = 0.37 [*P* = .01]) and casein-specific IgE titer (*r*_*s*_ = 0.13 [*P* = .39]) were not correlated with mean wheal diameter. However, casein-specific IgG4 titer (*r*_*s*_ = –0.51 [*P* < .0001]) and casein-specific IgG4–to–casein-specific IgE ratio (*r*_*s*_ = –0.53 [*P* < .0001]) were negatively correlated with mean wheal diameter.

The effect of early and regular CMF ingestion on CMA prevention was previously demonstrated.^1^ The present SPADE study subgroup analysis, which was based on daily CMF consumption records, examined the mechanism of CMA prevention through early and regular CMF ingestion by analyzing cow’s milk protein–specific immunoglobulin production among cow’s milk–sensitized infants. Infants who continued daily and intermittent CMF ingestion up to age 6 months had a higher titer of casein-specific IgG4 than did those who discontinued CMF ingestion before age 6 months. Although IgG4 antibodies can be transferred transplacentally, they generally disappear before age 6 months.^3^ Therefore, we were able to measure titers of serum casein–specific IgG4 produced by the infants themselves, which would be influenced by their feeding patterns. Notably, both the frequency and volume of CMF ingestion were positively correlated with casein-specific IgG4 titer. This supports our previous findings that early CMF discontinuation increases the risk of CMA.^2^ In addition, the continuous group participants who were originally allocated to the SPADE study’s ingestion group had a higher casein-specific IgG4 titer than did those from the avoidance group. This suggests that it may be effective to promote casein-specific IgG4 production between 1 and 2 months of age, but further analyses are needed to explore this relationship.

Our results showed that the casein-specific IgG4 titer and casein-specific IgG4–to–casein-specific IgE ratio were significantly higher in participants without CMA at age 6 months, which corroborates the results of a previous study on cow’s milk oral immunotherapy in children.^4^ These findings indicate that continuous CMF ingestion promotes casein-specific IgG4 production and the acquisition of tolerance to cow’s milk protein among cow’s milk–sensitized infants. However, further examination is needed to determine whether casein-specific IgG4 protects against CMA development in early infancy. For example, our results found no difference in the serum titer of casein-specific IgG4 nor in the casein-specific IgG4–to casein-specific–IgE ratio between participants with and without CMA when divided into the continuous and discontinued groups. In contrast, the participants without CMA in the discontinued group had a significantly higher frequency and volume of CMF ingestion until CMF discontinuation than did the participants with CMA. It is possible that cow’s milk protein–specific IgG1, which plays a protective role against CMA development, is produced earlier than is cow’s milk protein–specific IgG4 through continuous CMF ingestion.^5^^,^^6^

This study had several limitations. First, our relatively small sample may have affected the statistical analyses (eg, lack of significant difference in casein-specific IgG4–to–casein-specific IgE ratio between participants with and without CMA). Second, we did not measure the cow’s milk protein concentration or cow’s milk protein–specific IgA and IgG4 levels in maternal breast milk, which may be associated with CMA development in infants.^7^ Third, we could not measure β-lactoglobulin–specific IgE or α-lactalbumin–specific IgE levels, as these were not included in the SPADE study. Evaluating these immunoglobulins may provide greater insight into the immunologic response to CMF ingestion. Fourth, blood samples were drawn only from participants with positive reactions to the cow’s milk SPT. Therefore, we could not evaluate the immunoglobulin titers in participants with a negative SPT response to cow’s milk. Accordingly, our findings do not provide insight into the relationship between continuous CMF ingestion and immunoglobulin production in nonsensitized infants. Our *post hoc* analysis showed that the cow’s milk–specific IgE and casein-specific IgE titers were not correlated with mean wheal diameter in cow’s milk SPTs. Some of the participants with elevated cow’s milk–specific IgE or casein-specific IgE titers may have had a negative SPT response to cow’s milk. However, for identifying sensitization in early infancy, the SPT may be more sensitive than analyses of allergen-specific IgE levels.^8^ Next, our analysis showed that the casein-specific IgG4 titer was negatively correlated with mean wheal diameter in cow’s milk SPTs. This suggests that even if the participants had elevated cow’s milk–specific IgE or casein-specific IgE titers, the higher titer of casein-specific IgG4 may suppress a positive SPT response to cow’s milk.

In conclusion, this analysis demonstrated that cow’s milk–sensitized infants who continued CMF ingestion had a higher casein-specific IgG4 titer than those who discontinued ingestion. Further randomized controlled trials are needed to determine whether continuous CMF ingestion promotes the acquisition of cow’s milk protein tolerance through cow’s milk protein–specific IgG4 production.

## Disclosure statement

Disclosure of potential conflict of interest: The authors declare that they have no relevant conflicts of interest.

**Clinical implications**

Cow’s milk–sensitized infants who continued daily or intermittent CMF ingestion up to age 6 months had a higher casein-specific IgG4 titer than did those who discontinued ingestion before age 6 months.

## Uncited Table

Table II

## Supplementary data

Supplementary data

Supplementary Figure s
