# Supplementary material for: Continuous cow’s milk protein ingestion during infancy may promote casein-specific IgG4 production
Source: J Allergy Clin Immunol Glob. 2024 Apr 10;3(3):100257. doi: 10.1016/j.jacig.2024.100257 (PMC11067534; doi:10.1016/j.jacig.2024.100257)
Supplement: Supplementary data [file mmc1.docx]

# Online Repository

# METHODS

## The SPADE study design

The SPADE study was a multicenter, open-label randomized controlled trial conducted on a birth cohort of newborns from 4 hospitals in Okinawa, Japan.^E1^ During that trial, participants ingested cow’s milk formula (CMF) as required to supplement breastfeeding until 1 month of age, whereupon they were randomly assigned to either a CMF ingestion group (intervention: daily CMF ingestion of ≥10 mL) or an avoidance group (CMF avoidance). This intervention was performed between 1 and 2 months of age. After reaching 3 months of age, participants ingested CMF on demand to supplement breastfeeding. At 3 months and 6 months of age, skin prick tests (SPTs) and open oral food challenges (OFCs) were performed to assess cow’s milk sensitization and cow’s milk allergy (CMA) development, respectively.

## Skin prick tests

SPTs were performed using standard techniques with commercial allergen extracts of cow’s milk, egg white, wheat, and soy (Torii Pharmaceutical Co. Ltd., Tokyo, Japan). The positive and negative controls were histamine dihydrochloride (10 mg/mL) and 50% glycerol solution, respectively. A mean wheal diameter ≥3 mm larger than the negative control or larger than half of the positive control was considered a positive response for sensitization. Blood samples were drawn from participants with positive responses to the cow’s milk SPT.

## Oral food challenges

The first OFC to CMF was performed in both the CMF ingestion group and avoidance group at 3 months of age. This OFC involved a cumulative dose of 50 mL of CMF (equivalent to 750 mg of cow’s milk protein). Participants with a positive response to the preceding cow’s milk SPT ingested 5 mL, 15 mL, and 30 mL of CMF at 30-minute intervals. Those with a negative response to the cow’s milk SPT ingested 50 mL of CMF in a single dose. The second OFC was performed in both groups at 6 months of age. This OFC involved a cumulative dose of 100 mL of CMF (equivalent to 1500 mg of cow’s milk protein). Participants with a positive response to the preceding cow’s milk SPT ingested 5 mL, 15 mL, 30 mL, and 50 mL of CMF at 30-minute intervals. Those with a negative reaction to the cow’s milk SPT ingested 100 mL of CMF in a single dose. The OFC results were interpreted in accordance with the Japanese Guidelines for Food Allergy 2017 criteria.^E2^ Specifically, participants were diagnosed with CMA if they exhibited the following objective clinical reactions: urticaria, angioedema, vomiting, diarrhea, continuous cough, wheezing, stridor, or decreased blood pressure. Participants with a positive first OFC result continued in the study, and underwent the second OFC at 6 months of age. In addition, the intake of dairy products in withdrawers at 6 months of age was confirmed through phone interviews with their parents or guardians. Participants who exhibited allergic symptoms after ingesting dairy products were urged to visit a physician for examination and treatment.

## Immune parameters

Using the blood samples drawn from participants with positive responses to the cow’s milk SPT, we measured serum titers of cow’s milk-specific IgE (sIgE), casein-sIgE, and casein-sIgG4 using the ImmunoCAP system (Thermo Fisher Diagnostics KK, Tokyo, Japan).

## Statistical analysis

Participants who had ingested CMF in the first 3 days of life and exhibited a positive SPT response to cow’s milk at 6 months of age were selected for this subgroup analysis. Participants who continued CMF ingestion which include both of daily or intermittent ingestion up to 6 months of age were assigned to the continuous group, and participants who discontinued CMF ingestion before 6 months of age were assigned to the discontinued group (Figure E1 and Table E1).

The differences in cow’s milk-specific IgE, casein-sIgE, and casein-sIgG4 between the continuous group and the discontinued group were compared using the Mann-Whitney *U* test. To evaluate the influence of CMF ingestion frequency and volume, we used Spearman’s rank correlation coefficient to assess the correlations of the (a) total number of CMF ingestion days and (b) total volume of CMF ingestion quantity until 6 months of age with the 3 immunoglobulin titers.

Statistical significance was set at *P*<.05 (two-tailed), and analyses were performed using EZR software (Saitama Medical Center, Jichi Medical University, Saitama, Japan).^E3^ Each participant’s parent/guardian provided written informed consent upon recruitment, and the study was approved by each participating hospital’s institutional review board.

# References

E1. Sakihara T, Otsuji K, Arakaki Y, Hamada K, Sugiura S, Ito K. Randomized trial of early infant formula introduction to prevent cow's milk allergy. J Allergy Clin Immunol. 2021; 147: 224-232.e8.

E2. Ebisawa M, Ito K, Fujisawa T. Japanese Guidelines for Food Allergy 2017. Allergol Int. 2017; 66:248-264.

E3. Kanda Y. Investigation of the freely available easy-to-use software ‘EZR’ for medical statistics. Bone Marrow Transplant 2013; 48: 452-458.

**Table E1. Demographic and other characteristics of the participants**

|  | Continuous group  (n=21) | | Discontinued group  (n=28) | | *P* value |
| --- | --- | --- | --- | --- | --- |
| Male | 13 | (61.9) | 14 | (50.0) | .38 |
| Caesarean section | 1 | (4.8) | 8 | (28.6) | .06 |
| Season of birth: spring/summer | 11 | (52.4) | 18 | (64.3) | .56 |
| Gestational age (weeks) | 39 | (38 - 39) | 39 | (38 - 40) | .10 |
| Birth weight (g) | 3046 | (2795 - 3268) | 3091 | (2888 - 3269) | .94 |
| No siblings (first child) | 8 | (38.1) | 12 | (42.9) | >.99 |
| Maternal age | 31.5 | (27.5 – 35.5) | 32.0 | (30.8 – 37.0) | .24 |
| Maternal body mass index before pregnancy | 20.9 | (18.5 – 22.9) | 21.3 | (20.4 – 22.8) | .49 |
| Maternal weight gain during pregnancy | 10.4 | (7.6 – 13.1) | 10.4 | (8.0 – 11.7) | .58 |
| Maternal atopic diseases^a^ | 10 | (47.6) | 16 | (57.1) | .77 |
| Maternal smoking | 1 | (4.8) | 0 | (0) | .42 |
| Paternal age | 32.0 | (28.5 – 37.5) | 33.5 | (30.0 – 37.0) | .66 |
| Paternal atopic diseases^a^ | 13 | (61.9) | 10 | (35.7) | .04 |
| Paternal smoking | 8 | (38.1) | 6 | (21.4) | .20 |
| Domestic dog exposure at birth | 2 | (9.5) | 3 | (10.7) | >.99 |
| Domestic cat exposure at birth | 0 | (0) | 1 | (3.6) | >.99 |
| Maternal antibiotic use for treatment of chorioamnionitis and/or prevention of GBS infection^b^ | 2 | (9.5) | 1 | (3.6) | .57 |
| Neonatal antibiotic use^c^ | 2 | (9.5) | 2 | (7.3) | >.99 |
| Allocation to the SPADE study’s CMF ingestion group at age 1 mo | 7 | (33.3) | 4 | (14.3) | .16 |
| Eczema before age 3 mo | 15 | (71.4) | 13 | (46.4) | .08 |
| Eczema between age 3 and 5 mo | 10 | (47.6) | 11 | (39.3) | .56 |
| Continuation of breastfeeding up to age 6 mo | 12 | (57.1) | 28 | (100) | .0001 |
| Positive SPT response to egg white at age 6 mo | 10 | (47.6) | 12 | (42.9) | .77 |
| Mean diameter of wheals formed during the egg white SPTs at age 6 mo (mm) | 1.0 | (0.0 – 9.0) | 0.0 | (0.0 – 6.5) | .27 |
| Positive SPT response to wheat at age 6 mo | 2 | (9.5) | 0 | (0.0) | .17 |
| Mean diameter of wheals formed during the wheat SPTs at age 6 mo (mm) | 0.0 | (0.0 – 0.0) | 0.0 | (0.0 – 0.0) | .10 |
| Positive SPT response to soy at age 6 mo | 1 | (4.8) | 0 | (0.0) | .42 |
| Mean diameter of wheals formed during the soy SPTs at age 6 mo (mm) | 0.0 | (0.0 – 0.0) | 0.0 | (0.0 – 0.0) | .25 |

*Note:* Continuous group: Continued CMF ingestion up to 6 months of age; Discontinued group: Discontinued CMF ingestion before 6 months of age. Values are presented as number (percentage) or median (interquartile range). *P* values were calculated using Fisher’s exact test for categorical variables and the Mann-Whitney *U* test for continuous variables.

^a^ Atopic diseases include food allergy, bronchial asthma, atopic dermatitis, and allergic rhinitis.

^b^ Intrapartum antibiotic treatment for chorioamnionitis or intrapartum antibiotic prophylaxis to prevent the vertical transmission of GBS.

^c^ Antibiotic use in newborns within the first 7 days of life.

CMF, cow’s milk formula; GBS, Group B Streptococcus; SPADE, Strategy for Prevention of Milk Allergy by Daily Ingestion of Infant Formula in Early Infancy; SPT, skin prick test.

**Table E2. Clinical details of participants with positive results in the oral food challenge to CMF**

| No. | Group allocation  (SPADE study allocation) | Cumulative CMF (CMP) dosage, ml (mg) | Symptoms  (Medication) | Cow’s milk-specific IgE (kUA/L) | Casein-specific IgE (kUA/L) | Casein-specific IgG4 (mg_A_/L) | Reasons for discontinuing CMF ingestion |
| --- | --- | --- | --- | --- | --- | --- | --- |
| 1 | Continuous  (Avoidance) | 100 (1500) | LU (None) | 0.19 | 0.16 | 26.2 |  |
| 2 | Continuous  (Avoidance) | 100 (1500) | LU (None) | 1.04 | 0.37 | 0.25 |  |
| 3 | Discontinued  (Ingestion) | 100 (1500) | LU (None) | 0.78 | <0.10 | 0.09 | End of the designated ingestion period |
| 4 | Discontinued  (Ingestion) | 5 (75) | LU (None) | 0.53 | <0.10 | <0.07 | Mother’s desire to resume exclusive breastfeeding |
| 5 | Discontinued  (Avoidance) | 50 (750) | LU (None) | 0.90 | <0.10 | 0.19 | Mother’s desire to resume exclusive breastfeeding |
| 6 | Discontinued  (Avoidance) | 100 (1500) | LU (None) | 1.09 | 0.30 | 0.13 | Infant’s refusal |
| 7 | Discontinued  (Avoidance) | 20 (300) | LU, V (None) | 0.51 | <0.10 | 0.13 | No perceived need to use CMF after the avoidance period |
| 8 | Discontinued  (Avoidance) | 5 (75) | SU (Antihistamine) | 5.04 | 5.19 | 0.13 | Mother’s desire to resume exclusive breastfeeding |
| 9 | Discontinued  (Avoidance) | 100 (1500) | LU (None) | 1.26 | <0.10 | 0.12 | Start of the designated avoidance period |
| 10 | Discontinued  (Avoidance) | 5 (75) | LU (None) | 0.48 | 0.42 | 0.09 | No perceived need to use CMF after the avoidance period |
| 11 | Discontinued  (Avoidance) | 5 (75) | LU (None) | 3.39 | 2.60 | 0.09 | No perceived need to use CMF after the avoidance period |
| 12 | Discontinued  (Avoidance) | 20 (300) | SU (Antihistamine) | 21.1 | 26.3 | 0.09 | Start of the designated avoidance period |
| 13 | Discontinued  (Avoidance) | 20 (300) | SU, V, coughing, wheezing  (Antihistamine, Salbutamol) | 20.2 | 5.19 | 0.09 | Mother’s desire to resume exclusive breastfeeding |
| 14 | Discontinued  (Avoidance) | 20 (300) | LU (None) | 0.57 | <0.10 | 0.08 | Start of the designated avoidance period |
| 15 | Discontinued  (Avoidance) | 20 (300) | LU (None) | 0.52 | 0.20 | 0.07 | Start of the designated avoidance period |
| 16 | Discontinued  (Avoidance) | 100 (1500) | LU (None) | 0.69 | <0.10 | <0.07 | Mother’s desire to resume exclusive breastfeeding |
| 17 | Discontinued  (Avoidance) | 50 (750) | LU (None) | 1.13 | <0.10 | <0.07 | Start of the designated avoidance period |
| 18 | Discontinued  (Avoidance) | 50 (750) | LU (None) | 3.64 | <0.10 | <0.07 | Mother’s desire to resume exclusive breastfeeding |
| 19 | Discontinued  (Avoidance) | 100 (1500) | LU (None) | 0.49 | 0.44 | NA | Infant’s refusal |

CMF, cow’s milk formula; CMP, cow’s milk protein; LU, localized urticaria referring to noncontact urticarial reactions; SU, systemic urticaria; V, vomiting.

# Figure legend

**Figure E1. Study design**

In the continuous group, all 7 participants from the SPADE study’s ingestion group continued CMF ingestion (median of the mean daily consumption volume: 85 mL, interquartile range [IQR]: 61–≥200 mL) during the intervention period (between 1 and 2 months of age). Despite being allocated to the SPADE study’s avoidance group, 12 participants ingested small amounts of CMF (median of the mean daily consumption volume: 3.0 mL, IQR: 0.7–4.9 mL) and the remaining 2 participants intermittently continued CMF ingestion (mean daily consumption volume of 105 mL and 163 mL, respectively) during the intervention period. In the discontinued group, 2 participants from the ingestion group ingested almost no CMF (mean daily consumption volume of 0 mL and 1.7 mL, respectively) during the intervention period. The remaining 2 participants ingested CMF (mean daily consumption volume of 53 mL and 56 mL, respectively) during the intervention period; however, they discontinued CMF after the intervention period. All 24 participants from the avoidance group ingested almost no CMF (median of the mean daily consumption volume: 0 mL, IQR: 0–0.6 mL) during the intervention period.

CMF, cow’s milk formula; OFC, oral food challenge; SPADE, Strategy for Prevention of Milk Allergy by Daily Ingestion of Infant Formula in Early Infancy; SPT, skin prick test.
