# Supplementary figures and images for: Continuous cow’s milk protein ingestion during infancy may promote casein-specific IgG4 production
Source: J Allergy Clin Immunol Glob. 2024 Apr 10;3(3):100257. doi: 10.1016/j.jacig.2024.100257 (PMC11067534; doi:10.1016/j.jacig.2024.100257)

## Slide 1
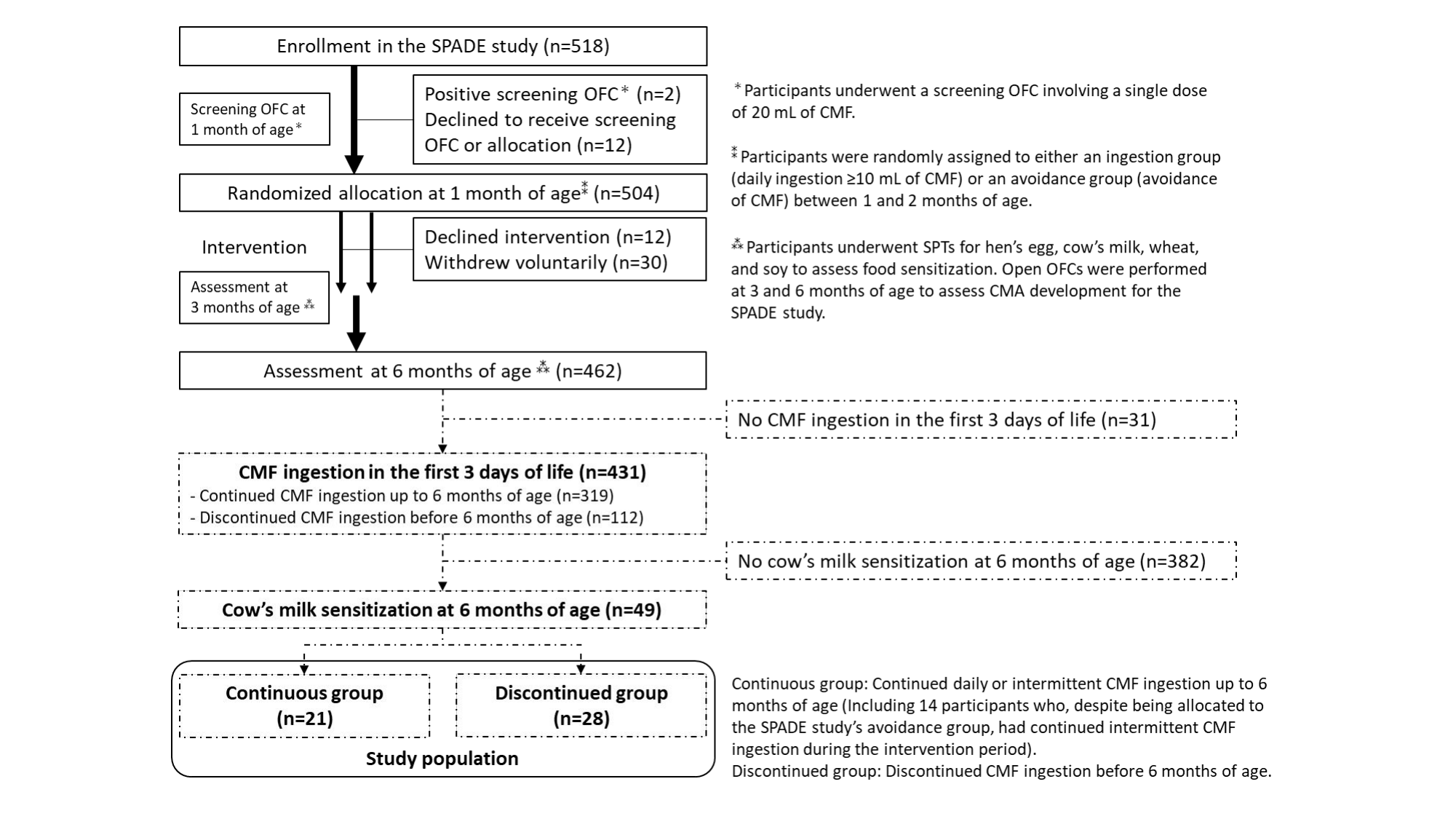

Supplement: Supplementary Figure — s [file mmc2.pptx]
